# Quantitative insights in tissue growth and morphogenesis with optogenetics

**DOI:** 10.1088/1478-3975/acf7a1

**Published:** 2023-09-28

**Authors:** Mayesha Sahir Mim, Caroline Knight, Jeremiah J Zartman

**Affiliations:** 1 Chemical and Biomolecular Engineering, University of Notre Dame, Notre Dame, IN 46556, United States of America

**Keywords:** optogenetics, morphogenesis, light-inducible, tissue engineering, signaling

## Abstract

Cells communicate with each other to jointly regulate cellular processes during cellular differentiation and tissue morphogenesis. This multiscale coordination arises through the spatiotemporal activity of morphogens to pattern cell signaling and transcriptional factor activity. This coded information controls cell mechanics, proliferation, and differentiation to shape the growth and morphogenesis of organs. While many of the molecular components and physical interactions have been identified in key model developmental systems, there are still many unresolved questions related to the dynamics involved due to challenges in precisely perturbing and quantitatively measuring signaling dynamics. Recently, a broad range of synthetic optogenetic tools have been developed and employed to quantitatively define relationships between signal transduction and downstream cellular responses. These optogenetic tools can control intracellular activities at the single cell or whole tissue scale to direct subsequent biological processes. In this brief review, we highlight a selected set of studies that develop and implement optogenetic tools to unravel quantitative biophysical mechanisms for tissue growth and morphogenesis across a broad range of biological systems through the manipulation of morphogens, signal transduction cascades, and cell mechanics. More generally, we discuss how optogenetic tools have emerged as a powerful platform for probing and controlling multicellular development.

**Glossary of abbreviations and acronyms**
*A*–*P* axis: Anterior–posterior axis
Bcd: Bicoid
BMP: Bone morphogenetic protein
Ca^2+^
: Calcium ion
CIBN: Cryptochrome-interacting bHLH N-terminal
CRAC: Ca^2+^ release-activated Ca^2+^
CRISPR: Clustered regularly interspaced short palindromic repeats
Cas9: CRISPR-associated protein 9 (Cas9)
CRY2: Cryptochrome 2
ERK: Extracellular signal related kinase
FGFR: Fibroblast growth factor receptor
GAP: GTPase activating protein
GEF: Guanine nucleotide exchange factor
Gt: Giant
GFP: Green fluorescent protein
Hkb: Huckebein
hPSC: Human pluripotent stem cell
hb: Hunchback
iLID: Improved light-induced dimer
kni: Knirps
Kr: Krüppel
LAVAs: Light activation at variable amplitudes
LED: Light-emitting diode
LEXY: Light-inducible nuclear export system
LITOS: LED illumination tool for optogenetic stimulation
LOV: Light-oxygen-voltage
AsLOV2: LOV2 domain of *Avena sativa* phototropin1
MDCK: Madin-Darby canine kidney
MCP: MS2-coat protein
MYPT: Myosin phosphatase target
NMII: Non-muscle myosin II
NES: Nuclear export sequence
PHYB: Phytochrome B
PI3K: Phosphoinositide 3-kinases
Raf: Rapidly accelerated fibrosarcoma
ROCK: Rho-associated protein kinase
SD1, or SD2: Shroom domain 1, or 2
SHH: Sonic Hedgehog
SOS: Son of sevenless
sspB: Stringent starvation *protein* B
tll: Tailless
tor: Torso
tsl: Torso-like
Trunk: trk
VEGF: Vascular endothelial growth Factor
WT: Wild type
Wnt: Wingless-related integration site
YAP: Yes-associated protein

## 1. Introduction

Robust organogenesis requires the integration of multiple cellular processes including cell proliferation, apoptosis, and differentiation, among others [1, 2]. This coordination depends on inductive signals called morphogens to control the differentiation state of cells and the shape of tissues [3]. The spatiotemporal distribution of morphogens provides positional information that determines cell identity across undifferentiated cellular fields [4, 5]. In turn, multicellular interactions that occur through chemical transport and reactions, mechanical forces, and electrical fields lead to the generation of tissue-level deformations such as the folding of epithelial layers into furrows or cups, the contraction or relaxation of cellular layers, or the exchange of cellular neighbors to elongate or migrate [6–16]. Positional information from morphogen gradients [17] is encoded and decoded by cell signaling networks to regulate the transcription of specific downstream genes and proteins to control cellular properties such as differential cell adhesion, cell motility, cell-matrix interactions, and cytoskeletal organization [2].

Defining the multimodal, multiscalar, and biophysical nature of organogenesis remains an unsolved mystery for physical and systems biologists to uncover [14]. Identifying the rules of morphogenesis remains a daunting task as it is a four-dimensional process involving time-dependent, complex 3D shaping of tissues and organs [2]. Traditional genetic perturbation and pharmacological studies often lack the high spatiotemporal resolution necessary to capture the dynamics of morphogen interactions in restricted cellular space and time windows. Hence, many biological processes in development and morphogenesis remain poorly understood and difficult to study using traditional tools.

Recently, optogenetics has emerged as a rapidly-expanding solution to better decipher biological processes as it leverages engineered light-sensitive protein constructs to control cells and their governing biomolecular processes and signaling pathways [18–20]. Around 1979, Francis Crick envisioned using a light-based technology to precisely control neuronal activity, but at the time, there were no methods to make neurons responsive to light [21]. Optogenetics was first implemented in the early 2000s by Deisseroth *et al* when they expressed light-sensitive microbial opsin proteins in neurons which made it possible to control the electrical activity of these neurons using light [18, 22, 23]. Initially, light-sensitive proteins, including ion channels or protein enzymes were used to control the activity of specific neurons in the brain, with the goal of understanding how these neurons contribute to brain function. Since its conception, optogenetic techniques have been increasingly used in neuroscience research to study the brain and its functions at the cellular level [19].

More recently, optogenetics is no longer limited to neurobiology and is increasingly being used across all areas of cell and developmental biology [24–26]. The true power of optogenetics lies in its unrivaled spatiotemporal resolution, with stimulation possible within a matter of milliseconds on a micrometer scale, to probe developmental processes and morphogenesis [27, 28]. This toolkit has emerged as an unmatched system to precisely probe the spatiotemporal mechanisms of cellular signaling networks and the mechanisms governing development [24, 29]. Optogenetics instantaneously, and reversibly with rare exceptions [30], activates a selective set of cells or tissue as opposed to non-selective activation of a larger region at low spatial resolution by chemical or electrical stimuli. It also consists of an extensive toolset that is cataloged by OptoBase [31]. A diversity of actuators and sensors exist [32] including plasma-membrane embedded channels, opsins like channelrhodopsin (ChR) or halorhodopsin, and photo-sensitive proteins that change conformation on activation, such as PHYB, CRY2, LOV domains, and fluorescent proteins like Dronpa [25, 33–49]. These light-sensitive actuators and proteins are used to engineer optogenetic constructs by manipulating their photosensitive domains.

For instance, the light-gated ion pore, ChR, is a microbial rhodopsin that helps sustain the survival and photosynthesis of unicellular motile algae by guiding it toward ambient light conditions [50]. Its chromophore, retinal, which covalently binds to opsin, remains in an all-trans/15-anti isomeric configuration in darkness but upon illumination, photon absorption triggers retinal isomerization and conversion to the 13-cis/15-syn retinal isomeric form occurs [51]. *In vivo* expression of ChR in the presence of all-trans retinal thus causes conformational changes in ChR leading to light-driven transportation of cations through the cell membrane [52]. Through iterative engineering efforts and innovation, many new and improved ChR variations have been synthesized. For example, Chrimson and Chronos were discovered through algal transcriptome sequencing and have further red-shifted excitation spectra and faster kinetics, respectively [53]. These sets of tools enable two-color activation in different regions of the same biological sample. Such engineered optogenetic tools have been deployed in systems ranging from cell-free to primates, and even in human clinical trials to bridge key knowledge gaps in biology [54–56].

Given the potential of optogenetic tools to advance investigations into cell and developmental biology, these tools are quickly creating new opportunities for rigorous quantitative studies This includes the ability to probe the dynamics of gene networks, expression patterns, and signaling cascades, which form the building blocks of complex morphogenetic processes [57]. For example, embryogenesis involves intricate gene networks, many of which are controlled by morphogens [58, 59]. Existing approaches can correlate certain genes or signaling dynamics with distinct developmental processes, but specific control of morphogen activity and the timeline of their regulatory processes are hard to decipher with genetic perturbation or mutagenesis. In contrast, optogenetics allows for the rapid actuation of morphogen activity, transcription factors, or genes with a short pulse of light at complex spatial patterns in embryos or tissues- providing a rapid and precise method for activating genes and measuring their real-time responses. When combined with other genetic perturbation techniques, this approach can activate cells or tissues with light, allowing for the quantification and characterization of expression outputs to elucidate detailed parameters of developmental and morphological processes [24, 60].

In this review, we focus on a selective subset of recent studies that have used optogenetics to generate specific quantitative insights into the biophysical mechanisms governing complex morphogenetic processes (box 1). While several comprehensive reviews have highlighted the utility of optogenetics to understand biological robustness in development [24, 26, 28, 60–62], this review also covers how optogenetics can modulate growth control. Further, we review examples that include post-embryonic development with a particular focus on highlighting the quantitative insights obtained using optogenetics, rather than the tools themselves. In the first section, we survey optogenetic tools that can control morphogen-regulated developmental processes and quantitative regulation of genes using such tools. Second, we highlight a few example studies on how crucial signaling dynamics can be programmed with optogenetics to define multiple cell fates within a tissue or to tune organ formation and growth control. Third, we compare studies striving to understand complex cellular mechanics by modulating cellular tension and force using optogenetic tools that regulate mechanical force generation. Of note, the full potential of optogenetics must still be combined with other complementary, advanced genetic techniques. In closing the review, we discuss the tremendous and growing potential of optogenetics to fill the knowledge gaps in morphogenesis and development at a more granular level of molecular and cellular activities by spatiotemporally controlling the dynamics of morphogens, gene networks, signaling cascades, and cell mechanics. In particular, we explore how optogenetics enables the systematic mapping of the limits of biological constraints, a key step in defining the contextual rules of life [63].

**Box 1.**
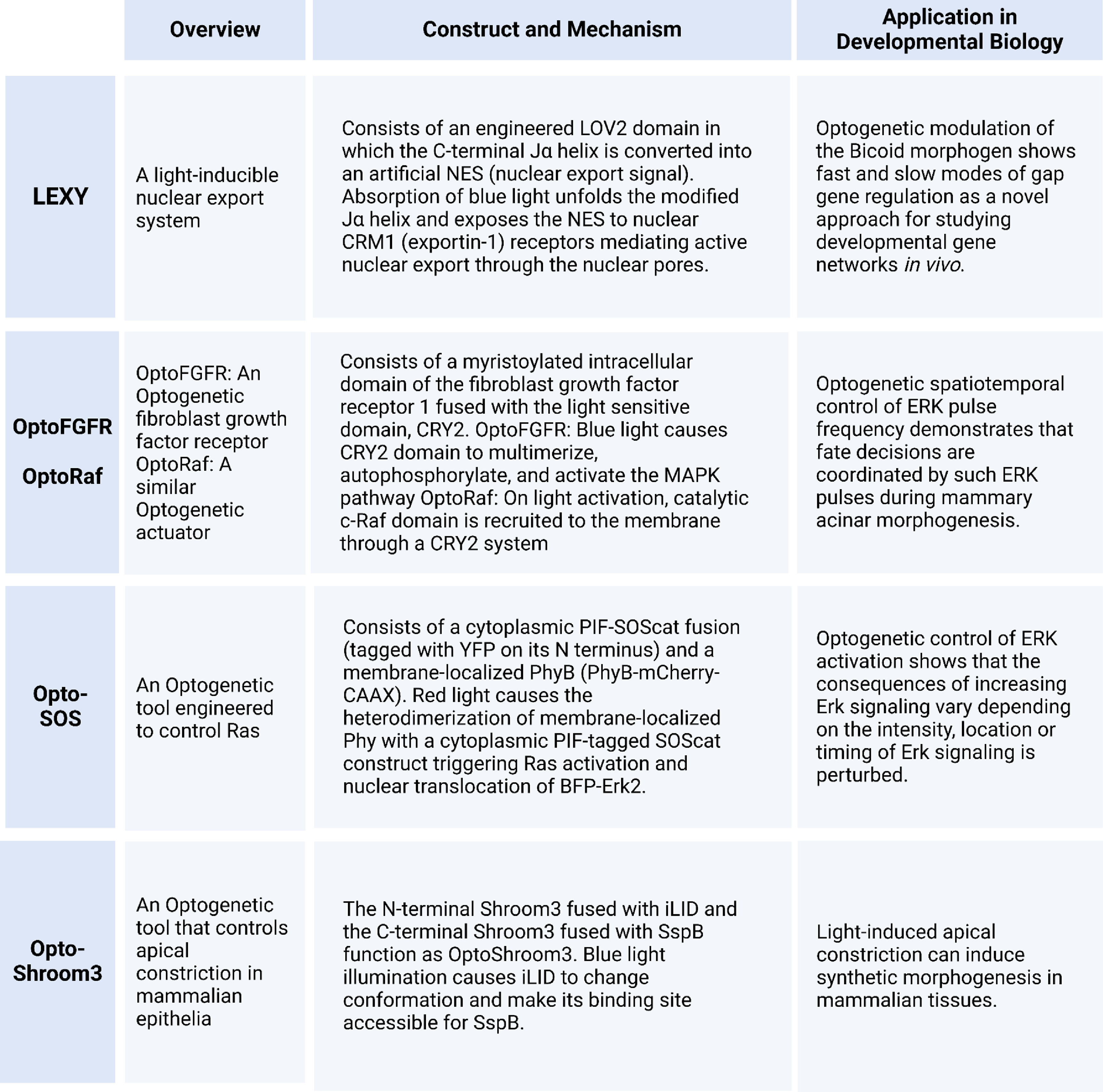
Example optogenetic tools highlighted in this review.

## 2. Measuring the specific dynamics of morphogen activity with optogenetics

**Figure 1.**
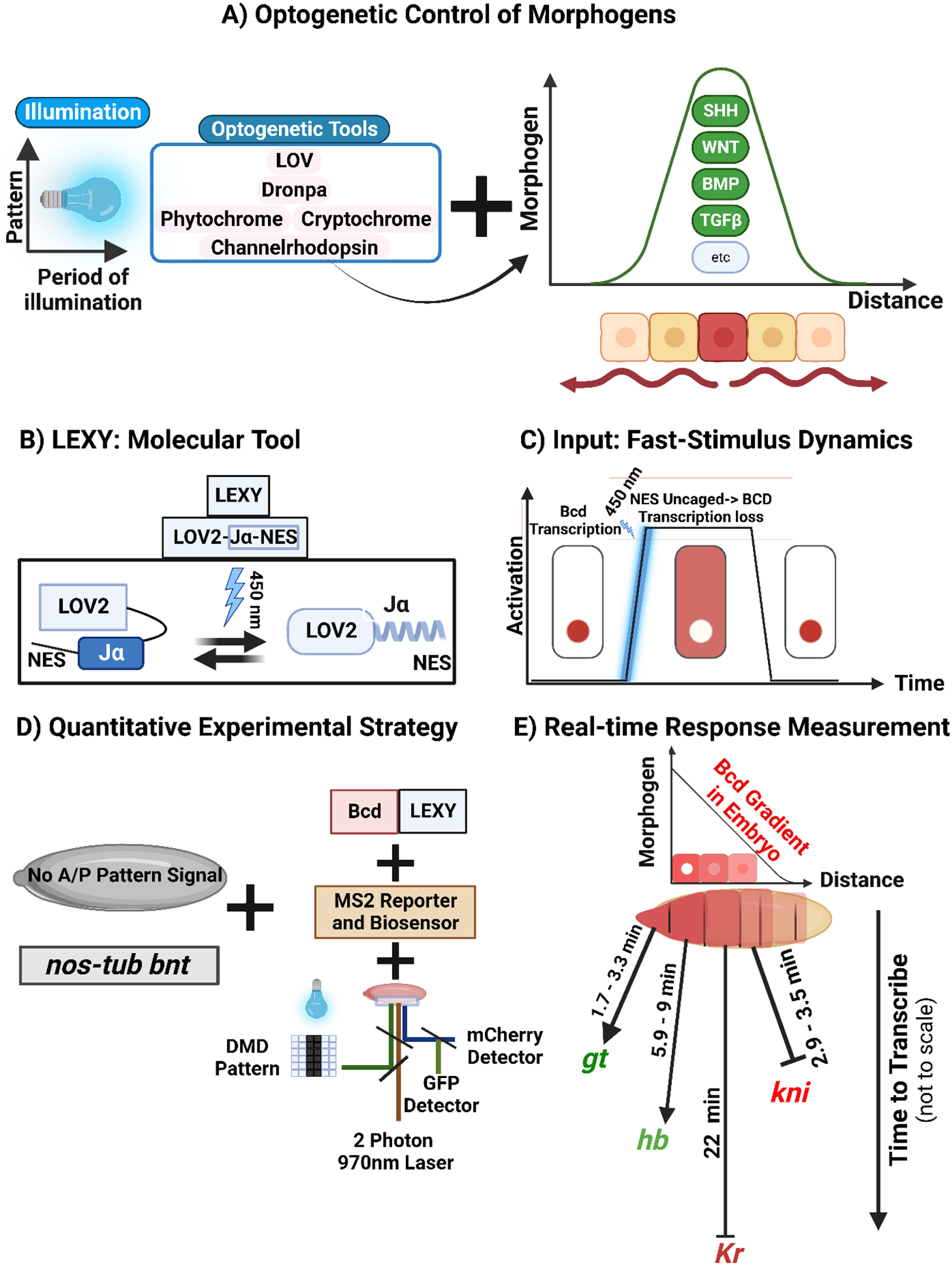
Optogenetic regulation of morphogen signaling activity can control gene expression and elucidate dynamics of downstream targets. (A) Various optogenetic domains and methodologies have been used to develop tools capable of regulating gradients of SHH, WNT, BMP, and other morphogens to study subsequent effects on development. Different illumination patterns and periods of activation may regulate morphogen secretion and signaling transduction in different manners. (B) As one example of optogenetic tools illuminating quantitative features of gene expression, the light-inducible nuclear export system, LEXY, whose C-terminal Jα helix contains a buried nuclear export sequence (NES) that is inactive in darkness but is uncaged upon activation with blue light, was utilized. (C) In this system, a pulse of a 450 nm blue light activates the nuclear export of Bicoid (Bcd), thus switching off transcriptional regulation by Bcd. (D) Mutant *bcd^E1^ nos^BN^ tsl^4^*/*bcd^E1^ nos^l7^ tsl^4^* (*bnt*) embryos lacking all three *A*–*P* patterning cues are combined with the Bcd-LEXY construct. The incorporation of MS2-based biosensors for each gap gene enables the quantification of gap gene transcription dynamics with a confocal two-photon imaging system. (E) Shifting from blue light to dark conditions increases Bcd activity resulting in a rapid increase of *gt* and *hb* gene expression in the anterior region of the mutant embryo while *kni* is rapidly repressed in the posterior region. With the optogenetic Bcd construct increasing due to a blue to dark shift, *Kr* was repressed in the medial region of the embryo with slower dynamics than the other gap genes. Created with BioRender.com.

Optogenetic methods are increasingly being used to regulate morphogen gradients and provide quantitative insights for various morphogen pathways (figure 1(A)). For example, a collection of optogenetic ePiggyBac vectors expressing a light-inducible split-Cre system with Cre-recombinase enzyme based on Magnets dimerization system was used to trigger *SHH* expression, allowing for the creation of spatially patterned neural cultures from stem cells, aligning with natural SHH gradients that shape the developing forebrain [64]. Similarly, a split CRISPR-Cas9-based photoactivatable transcription system, along with sgRNA for activating the *SHH* promoter, was used to locally induce and quantify increased *SHH* mRNA expression upon illumination in neuronal organoids to reveal its contribution to gene regulation in terms of the differentiation of neuronal progenitors, including the strong activation of IGF pathway modulators, the differentiation of cells expressing pericyte markers and the spatial modulation of axon guidance molecules. Programmable spatiotemporal patterns in a 3D neural organoid to study neurodevelopment were thus created using optogenetics [65].

As an example of a second optogenetic pathway tool, Opto-BMP activates BMP signaling through the interaction of the BMP kinase domains, as blue light-dimerizes the LOV domains fused to zebrafish BMP receptor kinase domains. Recorded transcriptional responses to optogenetic BMP signaling pulses demonstrate BMP signaling activation thresholds do not fully account for spatiotemporal gene expression. This further reveals that combinatorial signaling involving FGF and Nodal pathways generates spatial diversity in BMP-dependent gene expression in zebrafish [66]. A second tool for optogenetic control of BMP signaling with ‘OptoBMP’ has also been used to spatially coordinate the transformation of cardiac tissues and organoids through optically patterned differentiation [67].

As a third example pathway, Wnt/β-catenin signaling has been manipulated in hPSCs using a CRY2-based optogenetic tool called OptoWnt, which emulates the natural process of oligomerization of Wnt co-receptors upon the addition of Wnt ligand while allowing for on-cue control the Wnt signaling pathway using blue-light [68]. Light-based patterning of morphogen signaling using this tool enabled the robust, spatiotemporal differentiation of hPSCs into mesoderm, endothelial, and cardiac lineages [69]. This demonstrates how optogenetics can manipulate Wnt signaling [70] to induce stem cells to differentiate into cardiac cells, making it a tool for engineering tissues.

While the output of morphogen signaling results in spatial patterning of cell states, temporal dynamics are increasingly recognized as crucial for patterning. The temporal resolution afforded by optogenetic tools enables new insights into the temporal dynamics of signaling. As a key example, optogenetics enabled the control of morphogen-regulated spatial patterning in *Drosophila* embryo development to reveal the discovery and quantification of different speeds of regulation in gap genes [71]. In the *Drosophila* embryo, Bcd is a specialized morphogen that is also a key transcription factor that establishes the *A–P* axis of *Drosophila* embryos [72]. Bcd forms a concentration gradient from the head (anterior) to the tail (posterior) and activates or inhibits the transcription of gap genes to maintain the *A*–*P* axis of the embryo [73]. RNA binding protein, Nanos, and receptor tyrosine kinase (RTK), Torso also contribute to the establishment of the *A*–*P* axis. The combined gene network of Bcd, Nanos, and Torso forms a complex network that defines cell types along the *A*–*P* axis [74]. Recent efforts have used genetic engineering to eliminate all three of the patterning inputs to enable a platform for querying the specific individual functions of Bcd [75].

Singh *et al* revealed the dynamics of four Bcd-dependent gap gene regulations in the developing *Drosophila* embryo by optogenetically modulating the Bcd morphogen construct with LEXY (figure 1) [71]. They recapitulated rapid Bcd-dependent transcription of gap genes, *giant* (*gt*) and *hunchback* (*hb*), and delayed repression of *Krüppel* (*Kr*) previous demonstrated using traditional methods [76–78], and discovered a new role for Bcd in directly suppressing *knirps* (*kni*) transcription (figure 1(D)). This highlights the effectiveness of optogenetics for demonstrating causality [60]. LEXY, a reversible blue light-induced nuclear export system based on the AsLOV2 protein domain [79] was fused to Bcd to rapidly switch inputs to each gap gene. Under dark conditions, there is no NES activity, but blue light uncages the Jα helix to reveal the buried NES on the C-terminal of LEXY (figure 1(B)). This triggers the nuclear export of Bcd causing acute changes in nuclear Bcd concentration (figure 1(C)) [80]. A single-input system was created by expressing the Bcd-LEXY construct in mutant *bcd^E1^ nos^BN^ tsl^4^*/*bcd^E1^ nos^l7^ tsl^4^* (*bnt*) embryos lacking all three *A*–*P* patterning cues. For suppressing maternal Hb, a weak Nanos variant was expressed in the *bnt* mutant resulting in the combinatorial mutant *nos-tub bnt*. This mutant background was then combined with MS2-based transcriptional reporters to enable quantitative measurements of transcriptional activity for each gap gene in a ‘naive’ background state.

The imaging setup included a confocal microscope combined with a tunable two-photon (2P) laser to detect GFP and mCherry. A digital micromirror device with a blue LED enabled spatial patterning of optogenetic stimulation (figure 1(D)). Thus, the optogenetic rapid nuclear-cytosolic shuttling of Bcd was combined with the real-time readout of gap gene expression with fluorescent biosensors. The output recorded through MS2 spot quantification showed that the anteriorly expressed *gt* and *hb* responded within minutes to an increase in cytosolic Bcd concentration in darkness, but a slower and inverted response from the medial gap gene *Kr* implied indirect Bcd-induced repression. Of note, unexpected repression of the posteriorly expressed gap gene *kni* was observed- as fast *kni* transcription occurred upon acute loss of nuclear Bcd (figure 1(E)). This shows how optogenetics combined with previous genetic tools can be used to analyze gap gene networks to elucidate new insights into signaling dynamics and how a morphogen controls position-specific target gene expression as a function of specific developmental programs.

This optogenetic approach enables a fast and localized stimulus input to rapidly modulate morphogen activity. Combined with downstream fluorescent reporters, the subsequent gene dynamics can be quantitatively elucidated to provide new insights such as a comparison of quantitative response times. This approach can systematically be extended to define the dynamic relationships between other morphogens and downstream genes (figure 1(A)). For example, parallel studies are needed to investigate the gene networks of single-input Nanos and Torso in the embryo. Other key model systems such as the wing disc have benefited from the incorporation of the optogenetic ShineGal4, which has succeeded in elucidating morphogenetic steps of the wing formation from pupal wing to matured, adult wing [81]. A limitation of this approach is the need to create a genetic ‘blank state’ before introducing the artificial optogenetic morphogenetic stimulation tool. Whether non-reduced, i.e. wild-type systems expressing optogenetic tools can be utilized to infer similar insights through ectopic activation of pathway dynamics remains an open question. Computational modeling and design of experiment methodology may further enhance the utility of optogenetic studies to differentiate between competing mechanistic models [82, 83].

In sum, cellular pattern formation in tissues occurs through the arrangement of cells in an ordered, spatial configuration as directed by biological signals. Overall, optogenetic approaches in characterizing morphogens by light-mediated activation are rapidly developing and enable systematic quantification of morphogen-based signaling in developing and growing tissues. In the next section, we explore the optogenetically controllable features of these biological signals and how they relate to cell and tissue growth.

## 3. Defining the dynamic metrics of signal transduction with optogenetics to control growth and morphogenesis

**Figure 2.**
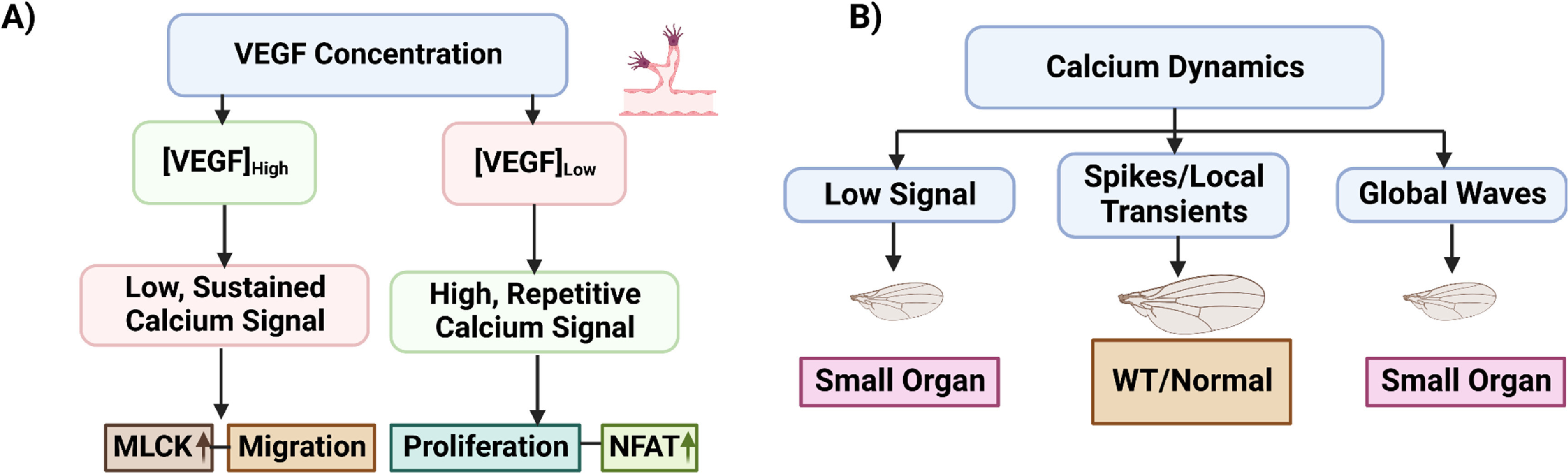
Modulation of morphogenesis and growth with different classes of multicellular signaling programs. (A) Ca^2+^ dynamics can control different outcomes during endothelial angiogenesis. VEGF-mediated Ca^2+^ dynamics dictate cell motility or division. Manipulation of VEGF concentration in endothelial cells during angiogenesis causes Low Persistent (LP) or repeated spikes (RS) Ca^2+^ waveforms. (B) A ‘Goldilocks Zone’ defines the biphasic regulation of key physiological pathways: Varying levels of integrated Ca^2+^ dynamics tunes organ size. In Drosophila melanogaster, genetic perturbation of cytosolic concentration of Ca^2+^ in wing primordium resulted in variation of the adult wing area.

Developmental patterns are vital to the proper growth of animals, but it is difficult to pinpoint the signaling parameters of patterns that bear crucial information in any given developmental context. Quantifiable features of a dynamic signal including amplitudes of concentrations, frequency, duration, delay, or area-under-curve [84] can dictate the fundamental cellular processes and cell fates, i.e. cell proliferation, survival, and apoptosis. For instance, VEGF-mediated Ca^2+^ signaling patterns, such as low, sustained signals and high, repetitive signals result in distinct functional responses during angiogenesis. These diverse responses include migration involving myosin light chain kinase (MLCK) and proliferation involving the transcription factor nuclear factor of activated T cells, respectively (figure 2(A)) [85]. Analogous to elusive downstream effects of ERK pulses, spatiotemporal dynamics of Ca^2+^ signaling in developing *Drosophila* wing discs have been categorized into four classes: single-cell spikes, intercellular transient, intercellular waves, and fluttering in the spatial range of tissue-level Ca^2+^ signaling in *Drosophila* wing morphology [86, 87]. Such Ca^2+^ dynamics may be similarly correlated in morphogenesis as varying Ca^2+^ plays the role of either a growth enhancer or suppressor and an optimal amount of Ca^2+^ signaling is required for robust size control. Low or globally high activities of calcium signaling reduced organ size whereas localized Ca^2+^ in cells induced larger organs indicating a ‘Goldilocks zone’ of integrated, optimal Ca^2+^ signaling (figure 2(B)). Optogenetic spatial and temporal control over Ca^2+^ signaling will allow for controlling the signaling cascade and cellular processes and can thus be utilized to similarly control size and growth control.

More generally, second messengers, such as calcium ions (Ca^2+^) or diacylglycerol regulate diverse cellular fates and growth-related outcomes [88–90]. Control over signal transducers such as second messengers can thus be used to pose new questions regarding information embedded in signaling features and to test a diverse range of temporal dynamics. In particular, Ca^2+^ is a versatile and ubiquitously present second messenger which profoundly controls cellular processes including proliferation and death [91, 92]. Recently, Lai *et al* [93] used R-GECO [94] and LAR-GECO1.2-mt [95] Ca^2+^ indicators co-transfected in calcium translocating channelrhodopsin (CatCh) [96] expressing U2OS-CatCh–Venus cells and activated CatCh with different illumination parameters including frequency, intensity and exposure time of light activation to program Ca^2+^ oscillation. Higher frequency of light activation of CatCh caused mitochondrial dysfunction, cytotoxicity, apoptosis, and triggered DRP1 (mitochondrial fission protein, dynamin-related protein 1) phosphorylation by the upstream Ca^2+^-dependent CaMKII, ERK1/2, and CDK1 pathways as the mitochondria were guided toward fission state with a significant decrease in mitochondrial size and increase in mitochondrial number.

As a vital dynamic signaling pathway, ERK plays crucial roles in many developmental processes including mammary gland development and primary mammary cell culture survival [97]. Yet, it has been difficult to define how specific ERK dynamics can govern such a large range of cellular processes robustly. As Ca^2+^ controls signaling pathways such as ERK to control growth [98], existing optogenetic tools controlling these pathways potentially can be used for controlling the growth and development of multicellular tissues through the recapitulation and reverse engineering of signaling dynamics.

**Figure 3.**
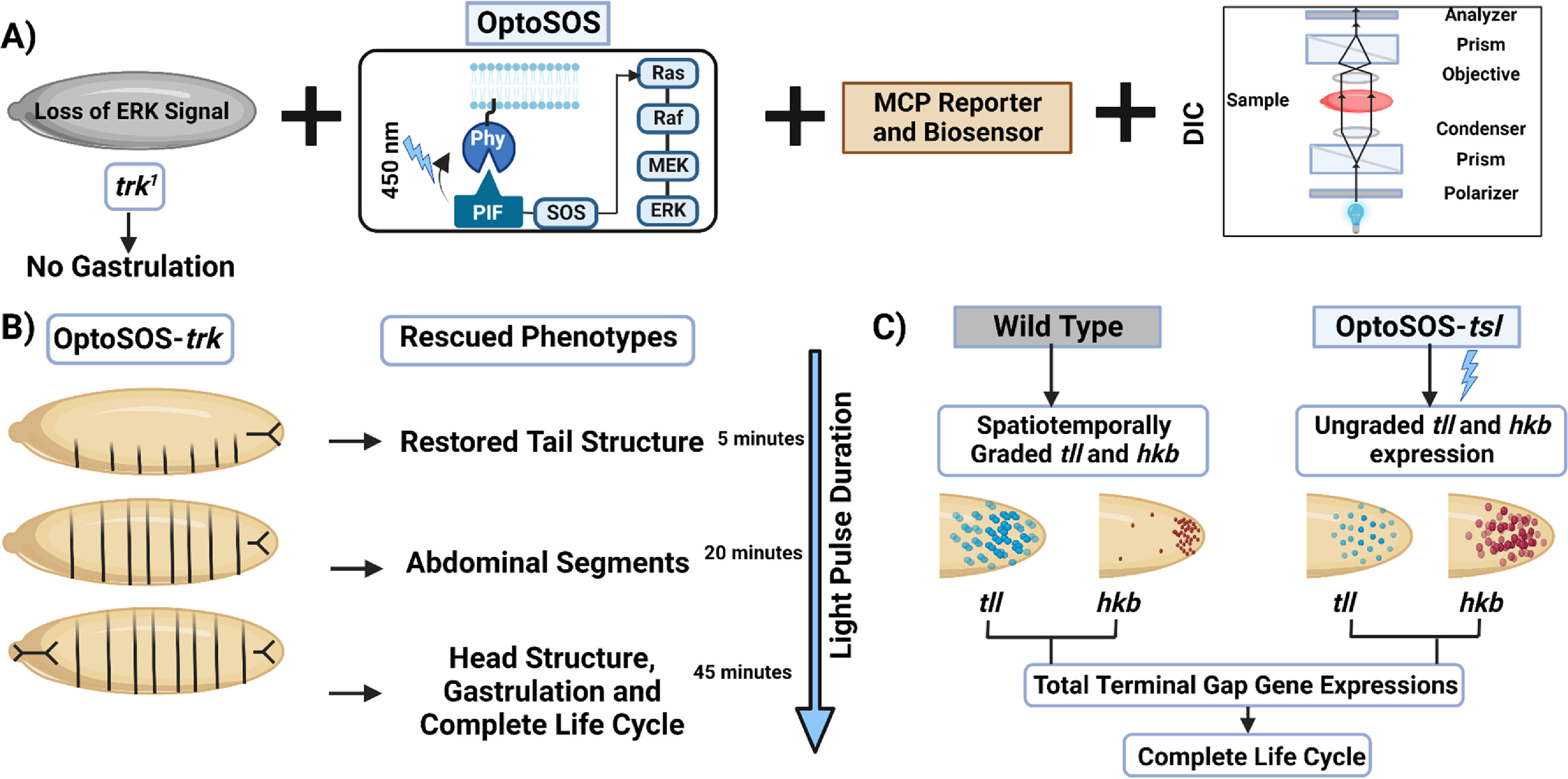
Simple switch-like optogenetic signals can replace signaling gradients. (A) Loss of function *trk* ^1^ mutant lacks Erk activity at the termini of the embryo. Opto-SOS as an optogenetic tool can be expressed in this mutant for gain-of-function of terminal signaling. Through the addition of biosensors and MCP (MS2 coating protein), the resulting outcomes can be imaged with Differential interference contrast (DIC) microscopy. (B) Distinct phenotypes can be rescued in the Opto-SOS expressed in *trk* embryos at increasing light thresholds in a switch-like manner. At 5 min of light pulses, the tail structure gets restored, while around 20 min, prominent abdominal segments arise, and finally, at 45 min of illumination, head structure and complete gastrulation is rescued. (C) Spatiotemporally graded and ungraded expressions are seen, respectively, in wild-type (OptoSOS) embryos and mutant OptoSOS-*tsl* embryos harboring MS2 stem loops driven by the *tll* or *hkb* upstream regulatory sequences.

Surprisingly, optogenetics demonstrates that a simple, all-or-none light input is sufficient to rescue a lethal mutant lacking terminal ERK signaling (figure 3) [99]. For optogenetic control of terminal ERK at the anterior and posterior ends of the embryo, Opto-SOS [100], an optogenetic system activating the Ras/Erk pathway downstream of RTKs can be combined with the genetic loss of terminal receptor-level components Trunk (Trk), Torso-like (Tsl), or Torso (Tor) (figure 3(A)) [100–102]. The *trk* ^1^ *Drosophila* mutant has a complete loss of terminal doubly phosphorylated Erk at the termini of the embryo. The progeny embryos of this mutant lack all endogenous terminal signaling activity in darkness while ERK is activated with exposure to 450 nm blue light. For recording spatiotemporal Erk signaling and gene expression, live-cell fluorescent biosensors were co-expressed with the Opto-SOS expressed in the *trk* mutants, and differential interference contrast (DIC) microscopy was employed to visualize the development of the embryos [103]. At temporally increasing light thresholds, strong gain-of-function terminal signaling rescued distinct phenotypes in a switch-like manner. All-or-none blue light pulses ranging from 5 to 45 min restored tail structure, embryo abdominal segments arise, head structure, and complete gastrulation leading up to adult flies that mate and lay eggs to complete a full life cycle (figure 3(B)). To identify the vital signaling parameters for maintaining the normal pattern of development, Erk-dependent target genes, *tll* and *hkb* were then monitored using upstream regulatory sequences that drove expression of MS2-tagged mRNAs combined with the fluorescent MCP expressed in OptoSOS-*tsl* mutants. Graded spatiotemporal expression of *tll* and *hkb* emerges during nuclear cycle progression in wild-type, but such graded expression of the genes is absent in optogenetically activated OptoSOS-*tsl* (figure 3(C)). However, the morphogenetic movements during gastrulation were robust to variations in the pattern width and timing, and optogenetically activated embryos without graded target gene expression undergo gastrulation and retain a full life cycle by emerging as adult flies and reproducing.

**Figure 4.**
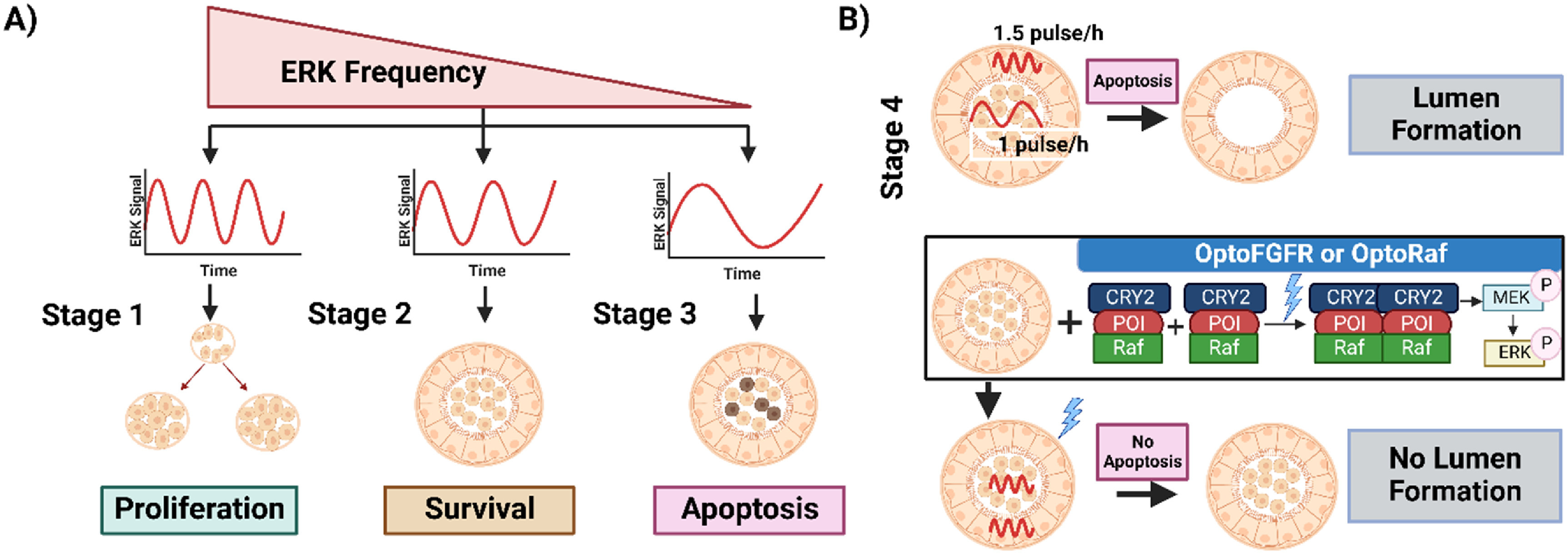
Optogenetic regulation of morphogenesis with varying signaling parameters. (A) Encoding multiple cell fates with signaling parameters: average frequency of non-periodic ERK pulses determines cell fate. During the development of mammary acinar, in early stage 1, high frequency of ERK induces cell proliferation. In contrast, a relatively slower ERK frequency ensures survival in the quiescent stage 2. In stage 3, low-frequency ERK in inner cells causes apoptosis and simultaneous higher frequency in the outer layer ensures survival leading to lumen formation. (B) Spatial compartmentalization deciphered using optogenetics: In stage 4, lumen formation occurs in wild type succeeding unsynchronized ERK frequencies in inner and out layers in stage 3. OptoFGFR and OptoRaf mediated synchronization of the ERK frequencies in outer and inner cells during the last stage of development prevented apoptosis of inner cells inhibiting natural lumen formation- which is similar to the dynamics of cancerous PIK3CA H1047R mutants which suffers from loss of lumen formation due to high ERK frequency.

As an example of this approach, Ender *et al* used optogenetics to provide new insights into the role of ERK signaling in mammary gland development and the importance of spatiotemporal control of signaling pathways in coordinating proliferation, survival, and apoptosis fates during development (figures 4(A) and (B)) [104]. This study concluded that fate decisions during acinar morphogenesis are coordinated by diverse spatiotemporal modalities of non-periodic ERK pulse frequency. Using a reporter cell line, mammary MCF10A cells producing acini, that expresses biosensors to track ERK activity, previously discovered proliferation, quiescence, apoptosis fates, and a mature lumen formation during acinar morphogenesis were recapitulated and were defined as stages 1–3 (figure 4(A)), and 4 (figure 4(B)), respectively [105, 106]. High-frequency, asynchronous ERK pulse in stage 1 is distinguished as the proliferative and highly motile stage, lower frequency in quiescent stage 2 has low motility and cell survival, while stage 3 characterizes the apoptotic fate of inner cells exhibiting low ERK frequency leading to the formation of a hollow lumen in stage 4 (figure 4(A)).

To prove the hypothesis that unsynchronized ERK pulses in inner and outer cell layers in stage 2 dictate cell survival or apoptosis, they evoked distinct frequency-modulated ERK pulses in acini by expressing two optogenetic Cry2-based blue light-activatable actuators: (1) optogenetic fibroblast growth factor receptor (optoFGFR) [107], a RTK that activates ERK, Akt, and Ca^2+^ signaling, (2) OptoRaf [108] which specifically activates ERK by recruiting a catalytic Raf domain to a plasma membrane-targeted anchor on blue light illumination. Using these two tools along with an LITOS as a light source were then used in quiescent Stage 2 [109]. This stimulated synchronized ERK frequencies in both inner and outer cell layers resulting in the survival of both the inner and outer cells with a failure to form a lumen as is seen in oncogenic PI3K signaling with aberrantly increased ERK frequency and proliferation (figure 4(B)). These experiments also found that cells in acini cannot survive unless they experience at least one ERK pulse every 4 h while causally connecting high ERK pulse frequency with inner cell survival. Optogenetic variation of the ERK pulse widths thus proved that ERK frequency alone regulates survival rather than integrated ERK activity.

In another recent study, strategic activation of OptoFGFR1 regulated ERK/AKT dynamics, unveiling the significance of temporal ERK activity as a critical factor in regulating pluripotency and the ability of mouse embryonic stem cells to maintain a memory of such signaling events [110]. These studies exemplify that many pathways encode key physiological information through pulses of transducers such as ERK and Ca^2+^. These examples highlight the power of quantitative mapping of cell signaling combined with optogenetics in substituting endogenous signals with externally controllable ones to manually pattern cellular processes. This not only enables studies that vary specific phenotypes but also provides insight into the parameters most important in a signal dynamic- such as the frequency of ERK pulses in mammary acinar morphogenesis.

Optogenetic actuators are also developed for other key growth pathways, including for Hippo/Yap. OptoYAP spatiotemporally controls the YAP/TAZ (Yes-associated protein and its homolog TAZ) pathway to control cellular proliferation and growth [111]. OptoYAP is shuttled to the nucleus through light activation as a LOV2 domain is fused to the N-terminus of YAP photocaging a buried nuclear localization signal (NLS) but the LOV2 domain unfolds and uncages the NLS upon illumination with 488 nm light. Specific control over the manipulation of YAP cellular localization using optoYAP has proven that its activation accelerates wound healing in cardiomyoblasts, introducing optogenetics as a probable tool to drive tissue regeneration [112]. Optogenetics can also regulate metabolism. As one example, engineered optogenetic photoreceptors have also emerged to regulate bacterial gene expression in *E. coli* to control mitochondrial dynamics, lifespan, and culture growth rate [113, 114].

A very interesting avenue in future research is to utilize optogenetics and other perturbation modalities to dissect the crosstalk between ERK, Ca^2+^, and Hippo signaling dynamics in these systems and to better understand how cell, tissue, and organ growth are controlled by such signaling dynamics and their interactions.

## 4. Probing tissue cell mechanics with optogenetics

How cells generate forces to cause cell shape changes and cell rearrangements that drive tissue morphogenesis is an important question in development. Contractility is a biological property caused by NMII, and it contributes to cellular processes, e.g. cytokinesis, cell motility, and cell shape or area changes [9]. NMII function is dependent on the Rho-kinase (ROCK) pathway and the phosphorylation of its subunit myosin light chain (MLC) by MLCK. Inhibition of inhibiting the phosphorylation of the MYPT subunit can lead to RhoA-ROCK signaling inactivating myosin light chain phosphatase (MLCP). MLCP consists of a regulatory subunit MYPT1, a catalytic subunit PP1c, and a small subunit M20 [115]. Consisting of myosin and actin, actomyosin is a contractile complex in which contraction occurs when the actin filaments are pulled by myosin motor proteins [116]. Actomyosin contractility causes cells to actively reduce their apical surface, which is necessary for the formation of numerous curved structures in metazoan embryos. Actomyosin contraction is triggered by the activation of the Rho-ROCK pathway on the apical side causing cells to reduce the surface area on its apical side- thus this process is called apical constriction [117, 118]. Yamamoto *et al* designed an OptoMYPT system activatable with blue light which relaxes cellular tension for reducing intracellular contractile force below the basal level [119]. Another optogenetic manipulation tool, MLCP-BcLOV4, was recently developed to study the morphogenesis of marine organisms by linking the PP1c-MYPT to the photosensitive LOV flavoprotein from *Botrytis cinerea* (BcLOV4) with an NES added to its C-terminus to regulate the localization activity and polarized distribution of NMII [120]. Activation of this optogenetic system abolished the apical contraction and led to the failure of epidermal cell invagination as the atrial siphon invagination was disrupted in *Ciona* larvae.

Using optogenetic technology to regulate the Rho signaling pathway has been a key method of reversibly manipulating cell mechanics with high spatiotemporal precision [121]. Activation of the small GTPase RhoA causes cellular contractility. Consequently, Rho1-activating optogenetic tools, such as optoGEF-RhoA [122], which uses localization of GEFs to control RhoA activity, have been engineered to modulate this process [122–124]. For instance, Rho1 activity can be up or down-regulated with optoGEF and optoGAP to regulate NMII concentration and localization and the perturbation of myosin gradients ultimately affects convergent extension during tissue elongation as myosin contracts in specific patterns to shape tissues [125]. Activation of optoGEF and optoGAP proved that mechanics of tissue deformation and flow through the organization of cell rearrangements aiding axis elongation, the regulation of cell area changes, and cell packings were all dependent on the Rho1 activity ranges, and the balance between medial and junctional myosin in *Drosophila* embryo.

Another photoswitchable tool to control RhoA is psRhoGEF [126], which has a GEF DH-PH domain that is anchored at the plasma membrane but is optically controlled by the photodissociable dimeric Dronpa. This tool was used to show that RhoA causes varying downstream effects and subsequent contrasting morphological alterations such as FA disassembly during cell retraction. Blue-light illumination causes accumulation of an NMII regulator at the cell membrane as induced by the dimerization of CIBN targeted to the cell membrane and CRY2 fused upstream of NMII regulators of the Rho/ROCK pathway in these optogenetic tools.

Such RhoA manipulation-based tools have helped quantify morphodynamic time scales, regions of local perturbations, and cellular forces, and provided detailed insights into morphogenetic processes like cytokinesis, assembly of contractile rings, or furrow formation [123]. Impacting the tissue microenvironment, Opto-RhoA-fibroblasts is a Cry2-CIBN-based optogenetic probe encapsulated within collagen for photoactivation of RhoA to induce local contractions within engineered 3D microtissues. This tool enables spatiotemporal control and quantification of the stress of specific regions of microtissues and inference of tissue strain using particle image velocimetry [127]. Heydasch *et al* engineered a genetic circuit to explore the mechanism of the Rho-specific GAP protein, Deleted in Liver Cancer 1 (DLC1) to control Rho GTPase signaling fluxes [128]. This circuit consisted of an iLID-based optogenetic actuator, optoLARG (optogenetic leukemia-associated Rho GEF), which is capable of transient optogenetic recruitment of a RhoGEF domain, and a spectrally compatible Rho activity biosensor to interrogate RhoGTPase flux. Acute optogenetic manipulation of local Rho activity and contractility revealed that DLC1 controls the rate of Rho activation rather than duration, both at focal adhesions and at the plasma membrane, and DLC1 associates/dissociates with focal adhesions during their mechanical reinforcement/relaxation.

The adaptability of these versatile optogenetics tools can thus lead to novel insights into robust development as well as disease-related phenotypes by precisely regulating contractile force generation.

**Figure 5.**
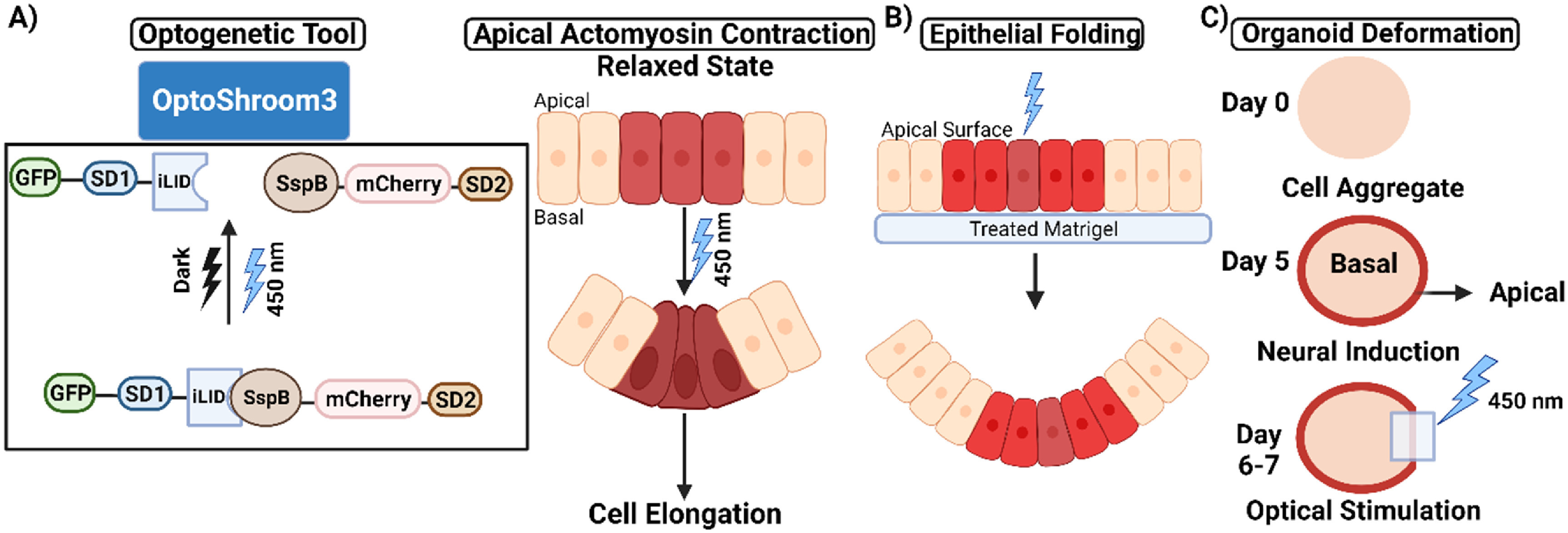
Inducing synthetic morphogenesis through optogenetic induction of apical constriction. (A) Optogenetic version of Shroom3: Shroom3 causes apical constriction and cell elongation by recruiting ROCK to apical junctions. (B) Optoshroom3-provoked apical constriction causes irreversible curvatures and folding of cell sheets in MDCK monolayer colonies on a soft gel. (C) Flattening caused by local optogenetic activation Optoshroom3 in neuroectodermal organoids.

The optogenetic tool, Optoshroom3, which rapidly and reversibly controls apical constriction, demonstrated that inducing apical constriction in different contexts can result in the folding of epithelial cell colonies on soft gels, thickening of neuroepithelia, apical lumen reduction in optic vesicles, and flattening in neuroectodermal tissues [129]. While it is used to manipulate the process of apical constriction similar to other optogenetic tools like OptoGEF-RhoA, OptoShroom3 is specific to the apical side of cells whereas the catalytic domain of the RhoA activator is fused to CRY2-mCherry in tools like OptoGEF-RhoA, and its binding partner CIBN to bind either to the plasma membrane or to the mitochondrial membrane [122, 129]. Actin-binding SD1 of Shroom3 regulates apical localization, and the SD2 is essential for the binding to ROCK [130]. Upon blue light illumination, an iLID, which controls the localization and activity of signaling proteins, changes conformation and makes its binding site accessible for SspB [131]. N-terminal Shroom3 fused with iLID and the C-terminal Shroom3 fused with SspB function as an optogenetic split-version of Shroom3, i.e. OptoShroom3. OptoShroom3 constructs reversibly dimerize upon blue light stimulation through light-induced binding of the iLID-SspB optogenetic pair (figure 5(A) Left). Redistribution of NMII in the apical junctions of MDCK cells resulted in OptoShroom3-induced apical constriction, which caused a subsequent reduction of the apical cell surface and cell elongation (figure 5(A) right). These observations with an assumption of a linear change in basal to apical cross-sectional area were matched to the following model for predicting cell height as:

\begin{align*} &amp; {\text{Predicted}}{\mkern 1mu} {\text{Cell}}{\mkern 1mu} {\text{Height}}\\ &amp; \quad = {\mkern 1mu} \left( {2*{\text{Measured}} {\text{Cell}} {\text{Volume}}} \right)/ \\ &amp; \qquad \left( {{\text{Measured}}\left( {{\text{Apical}}{\mkern 1mu} {\text{Area}} + {\text{Basal}}{\mkern 1mu} {\text{Area}}} \right)} \right){\mkern 1mu} \end{align*}

This simple model accurately matched the prediction with observations. This further supports the model that cell height increases for a reduced apical area with constant cell volume and basal area.

In other optogenetic experiments, light-stimulated apical constriction is shown to regulate morphogenetic tissue deformation of organoids to control epithelial cell colony folding on soft gels (figure 5(B)), flattening of neuroectodermal tissues (figure 5(C)), epithelial thickening of neuroepithelial layers, and lumen reduction in mouse optic vesicle organoid. These results prove the efficiency of optogenetics in spatiotemporal control of apical constriction and the resulting contextual versatility of 3D deformation depending on the initial tissue context and specific optogenetic program.

The wide array of optogenetic tools, combined with straightforward experimentation using established protocols, offers a promising opportunity to advance research in developmental biology by enabling precise manipulation of cellular mechanics [132]. However, a major challenge of using optogenetic tools to manipulate cellular mechanics is that most tools created to date rely on the activation of LOV2 and CRY2 by blue light. This makes the visualization of proteins in the widely utilized green channel unfeasible and impedes the observation of GFP-based markers [121]. Hence, there is a need for more innovation in optogenetic tools that may utilize other wavelength of light to manipulate cellular mechanics more efficiently.

## 5. Conclusions and translation into new applications

The highlighted studies in this brief review demonstrate how the rapidly increasing arsenal of optogenetic tools is providing new quantitative insights into morphogenesis [133]. Genetic manipulation and transgenic techniques allow optogenetic systems to be expressed in cell colonies, tissues, organoids, and in cell-free light-inducible *E. coli* promoter systems [54, 134]. Optogenetic tools have been utilized in a broad range of animals including fruit flies, mice, zebrafish, and primates, and human clinical trials [18, 54, 55, 71, 99, 129, 135, 136]. Novel optogenetic tools expressed in this diverse range of systems are also complemented with the co-expression of biosensors, and cutting-edge imaging systems giving rise to an immense potential for quantifiable discoveries in developmental biology.

For example, as seen in figure 2, quantitative studies connecting second messengers, such as Ca^2+^ to developmental states provide a foundation for future optogenetic experiments that test whether direct regulation of second messengers is sufficient to suppress, enhance or redirect morphogenetic phenotypes. These studies will require optogenetic tools, such as channelrhodopsins or genetically encoded Ca^2+^ actuators, or Opto-CRAC, a near infrared-stimulable optogenetic platform that selectively and reversibly photo-manipulates Ca^2+^ influx through the CRAC channel to regulate the function of non-excitable cells [137].

As another dimension in this field of research, optogenetic tools like ArchT spatiotemporally increase intracellular pH, inducing membrane ruffling and potentially controlling migration and polarization [138]. Precise genome editing using photoactivatable Cas9 and optogenetic CRISPRi used in controlling metabolic fluxes for growth or metabolite production present promising potential for quantitative studies in complex morphogenetic processes [139, 140].

Similarly, the optogenetic toolkit for controlling cellular mechanics and the cytoskeleton is rapidly expanding [119, 122–125, 129]. Synthetic morphogenesis for designing cellular shapes and structures can be achieved with optogenetics as demonstrated with OptoShroom3 to control contractility [100]. Additionally, optogenetic control of motor proteins such as engineered myosin motors that are able to walk along the actin cytoskeleton in living cells and regulate cargo transport inside cellular extensions enable control of neurite extension and filopodia with high spatiotemporal resolution [141]. More complex morphogenetic programs and more intricate synthetically engineered tissues or organoids soon may be realizable with optogenetics as simple epithelial folding and thickening have already been implemented [129, 134, 142].

Synergizing these optogenetic tools with biosensors, reporters, imaging modalities, and illumination devices facilitates the precise use of optogenetic tools. In the context of live cell imaging, CRISPR/Cas9 gene editing was used to create a cell line with myosin-2A labeled with GFP and mCherry by inserting knock-in fusion tags into the myosin-2A gene. This allowed for the demonstration that when the lamin A/C is depleted, there is an increase in the formation of NMII bipolar filaments [143]. It may be possible to express optogenetic tools to this system to control myosin-2A gene expression and subsequent cell morphogenesis using feedback and automated microscopy. Additionally, LITOS and iLED are noted illumination devices assisting in understanding morphogenesis [109, 131]. Cutting-edge devices like LAVAs control the dose intensity and location of light used to activate optogenetic tools [144].

Optogenetics has the potential to further deepen our understanding of morphogenesis and development not only at a molecular and cellular level but also in solving real-world problems. Aside from exploring comprehensive aspects of cellular and subcellular developmental processes, optogenetic techniques are being utilized for human disease treatments. An example of the apex of application of these discoveries is the use of an optogenetic tool, ChrimsonR, which is a light-sensitive protein that acts as a photoswitch to restore visual function in patients with the inherited disease, Retinitis Pigmentosa. Phase I/II PIONEER trial has shown positive early data in combating vision loss due to this disease [136, 145, 146]. Nascent research and disease modeling also is utilizing optogenetic approaches for neural, cardiac, and immunoregulatory diseases such as Parkinson’s Disease, epilepsy, Bradycardia, Tachycardia, fibrillation, and melanoma [147–154].

In terms of understanding and curing developmental and growth-related pathologies, great strides have been made in organoid engineering, tissue regeneration, etc. For example, Huang *et al* optogenetically activated the LIM Homeobox 8 (Lhx8) target gene to demonstrate the vital role of the gene in controlling complementary proliferation and osteogenic differentiation expressed in bone development [155–157]. As an optogenetic tool, FKF1 (flavin-binding, kelch repeat, f-box 1) receptor with a LOV domain temporally induced Lhx8 expression activatable with blue light. Consequently, it was discovered that early stage of induction with optical activation improves osteogenesis of bone marrow mesenchymal stem cells and further bone formation by these stem cells [158]. In another tool discussed earlier in this article, Hellwarth *et al* implemented a Cry2-based optogenetic platform, OptoWnt to induce robust endothelial cardiovascular cell differentiation and cardiac tissue patterning differentiation of hPSCs via light-control regulation of the Wnt/*β*-catenin pathway [39, 68, 69, 144]. These studies demonstrate that optogenetic tools like OptoWnt have massive potential both for the precise engineering of tissue morphogenesis and organ development from stem cells and for discovering insights into clinical research.

Despite its capabilities, optogenetic tools have limitations. *In vivo* experimentation with optogenetic tools typically requires invasive procedures including the injection of a viral vector for genetic expression or implantation of an optical electrode due to low penetrance of light into tissue [159]. Novel methodologies incorporating ultrasound technology and longer wavelengths of excitation are emerging to circumvent this limitation [160–162]. When investigating extracellular morphogen signaling or long-range transport within cells, the possibility of diffusion of photoactivated optogenetic modules may disrupt gradients and lead to unintended activation [24]. This can be circumvented by defining activation in a specific region while the other regions remain deactivated. However, optogenetics’ reliance on genetic modification makes it challenging for human-cell based developmental studies and its primary usage is most natural for genetic model organisms [163].

Optogenetic tools allow novel mechanisms to control complex spatially influenced processes that modulate morphogenesis over time. Specifically, existing tools have interrogated and quantified, among many other questions, how transcription factors are transcribed and the dynamics of gene expression, which parameters of signaling cascades drive robust morphogenesis, and how contractility controls initial tissue organization and eventual organogenesis. There are still many unanswered questions in the field of development. Clearly, optogenetics will keep shining light on the broader quantitative principles controlling multicellular development with unique opportunities to translate these findings into new therapies and applications.

## Data Availability

No new data were created or analysed in this study.
